# The autotaxin-lysophosphatidic acid–lysophosphatidic acid receptor cascade: proposal of a novel potential therapeutic target for treating glioblastoma multiforme

**DOI:** 10.1186/s12944-015-0059-5

**Published:** 2015-06-18

**Authors:** Sadaharu Tabuchi

**Affiliations:** Department of Neurosurgery, Tottori Prefectural Central Hospital, 730 Ezu, Tottori, 680-0901 Japan

**Keywords:** Autotaxin, Lysophosphatidic acid receptor, Cancer, Glioblastoma, Angiogenesis, Invasion, Migration, Antagonist, Inhibitor, Therapy

## Abstract

Glioblastoma multiforme (GBM) is the most malignant tumor of the central nervous system (CNS). Its prognosis is one of the worst among all cancer types, and it is considered a fatal malignancy, incurable with conventional therapeutic strategies. As the bioactive multifunctional lipid mediator lysophosphatidic acid (LPA) is well recognized to be involved in the tumorigenesis of cancers by acting on G-protein-coupled receptors, LPA receptor (LPAR) antagonists and LPA synthesis inhibitors have been proposed as promising drugs for cancer treatment. Six LPARs, named LPA_1–6_, are currently recognized. Among them, LPA_1_ is the dominant LPAR in the CNS and is highly expressed in GBM in combination with the overexpression of autotaxin (ATX), the enzyme (a phosphodiesterase, which is a potent cell motility-stimulating factor) that produces LPA.

Invasion is a defining hallmark of GBM. LPA is significantly related to cell adhesion, cell motility, and invasion through the Rho family GTPases Rho and Rac. LPA_1_ is responsible for LPA-driven cell motility, which is attenuated by LPA_4_. GBM is among the most vascular human tumors. Although anti-angiogenic therapy (through the inhibition of vascular endothelial growth factor (VEGF)) was established, sufficient results have not been obtained because of the increased invasiveness triggered by anti-angiogenesis. As both ATX and LPA play a significant role in angiogenesis, similar to VEGF, inhibition of the ATX/LPA axis may be beneficial as a two-pronged therapy that includes anti-angiogenic and anti-invasion therapy. Conventional approaches to GBM are predominantly directed at cell proliferation. Recurrent tumors regrow from cells that have invaded brain tissues and are less proliferative, and are thus quite resistant to conventional drugs and radiation, which preferentially kill rapidly proliferating cells. A novel approach that targets this invasive subpopulation of GBM cells may improve the prognosis of GBM. Patients with GBM that contacts the subventricular zone (SVZ) have decreased survival. A putative source of GBM cells is the SVZ, the largest area of neurogenesis in the adult human brain. GBM stem cells in the SVZ that are positive for the neural stem cell surface antigen CD133 are highly tumorigenic and enriched in recurrent GBM. LPA_1_ expression appears to be increased in these cells. Here, the author reviews research on the ATX/LPAR axis, focusing on GBM and an ATX/LPAR-targeted approach.

## Introduction

Glioblastoma multiforme (GBM) is the most highly malignant type of brain tumor. Despite the use of optimal treatments and an evolving standard of care (maximal safe resection with concurrent temozolomide (TMZ) chemotherapy and radiation therapy), the median survival of patients diagnosed with GBM is only 12 to 16 months [1]. GBM cells are highly motile and invade the normal brain parenchyma diffusely, resulting in poor prognosis [2]. Because the blood-brain barrier (BBB) is disrupted in GBM, some components in plasma or serum may be able to affect the cell motility of GBM [3]. The most plausible components are considered to be autotaxin (ATX) and lysophosphatidic acid (LPA). ATX is a potent cell motility-stimulating factor that is identical to lysophospholipase D and that produces a bioactive phospholipid, LPA, from lysophosphatidyl choline. ATX is overexpressed in GBM. In addition, LPA_1_, the LPA receptor (LPAR) responsible for LPA-driven cell motility, is predominantly expressed in GBM [4]. These important results suggest that the ATX/LPAR axis may be a target for GBM therapy. Here, the author reviews current results, focusing on the promising information on the ATX/LPA/LPAR cascade that may lead to amelioration of GBM.

## Review

### GBM and therapeutic difficulty

GBM is the most aggressive type of tumor of the central nervous system (CNS), and its prognosis is one of the worst among all cancer types. GBM has diffuse, invasive, and highly angiogenic characteristics, which result in a high recurrence rate. Following surgical resection, the current standard of therapy involves concurrent administration of the DNA alkylating agent TMZ with radiation, followed by adjuvant TMZ [1]. In addition to TMZ, use of another agent, implants of biodegradable polymers containing the alkylating agent carmustine, was approved for clinical use. Although, a phase III trial has suggested a modest survival benefit [5], the study had several methodological problems and resulted in frequent toxicities, such as brain edema, infection, and seizures. A direct comparison of carmustine with standard chemotherapy with TMZ is lacking [6]. For the treatment of recurrent GBM, none of the available salvage treatments has clearly shown improved survival [6]. The chemicals in this class of alkylating agents are highly mutagenic. This reveals another aspect of a number of anti-cancer treatments: in addition to their effects in reducing or eliminating tumors, X-rays and certain traditional cytotoxic agents are also carcinogenic, and their short-term success in producing clinical remission may be counterbalanced by the later appearance of independently arising, second-site tumors that are a consequence of their mutagenic action [7].

Thus, at present, GBM is considered a fatal malignancy that is incurable with conventional therapeutic strategies. Given the resistance of GBM to conventional therapeutic approaches, an urgent need exists to develop alternative strategies to complement or improve current approaches and improve long-term patient survival. Strategies under development include novel adjuvant chemotherapeutics to be combined with standard care, as well as novel molecularly targeted approaches against the tumor and its environment.

### LPA, LPA receptors, and GBM

LPA (1- or 2-acyl-*sn*-glycerol 3-phosphate) is one of the simplest natural phospholipids, consisting of a single fatty acyl chain, a glycerol backbone, and a free phosphate group. LPA is a major active constituent of serum, and unlike most other phospholipids, it is also water soluble [8]. LPA is a main membrane-derived multifunctional lipid mediator that is best known for its ability to stimulate proliferation, migration, and survival of many cell types, both normal and malignant [9]. LPA has been implicated in the pathogenesis of several conditions, including cancer [8], atherosclerosis and cardiovascular disease [10, 11], Alzheimer disease [12], psychiatric disorders such as schizophrenia and bipolar disorders [13, 14], ischemic cerebrovascular disease [15], and hydrocephalus [16].

LPA is a bioactive phospholipid that stimulates cell proliferation, migration, and survival by acting on its cognate G-protein-coupled receptor (GPCR). Aberrant LPA production, receptor expression, and signaling probably contribute to cancer initiation, progression, and metastasis [8]. Although LPA production may partially occur intracellularly, most LPA is produced extracellularly by secreted enzymes. Three pathways mediate the production of LPA: (1) cleavage of lysophophatidylcholine by lysophospholipase D (lysoPLD) in blood, (2) deacylation of phosphatidic acid by phospholipase A_2_ in inflammatory cells, activated platelets, endothelial cells, and cancer cells, and (3) non-enzymatic, mild oxidation of low-density lipoprotein [17, 18].

The primary molecular mechanism was reported in 1996 with the cloning of the first cognate receptor for LPA [19]. Currently, at least six different LPA receptors (LPARs; LPA_1_-LPA_6_) have been identified that share a common GPCR structure [20]. All six receptors are expressed throughout the body during development and adulthood in unique spatiotemporal patterns [21].

Based on the amino acid sequence, LPA_1_-LPA_3_ share 50 % homology and belong to the endothelial differentiation gene (Edg) family. Within the brain, LPA_1_ is the most highly expressed, although LPA_2_ and LPA_3_ are also present [20]. In 2003, Noguchi *et al.* successfully identified LPA_4_ (p2y_9_/GPR23) through ligand screening of orphan GPCRs sharing high amino acid sequence homology with the human platelet-activating factor receptor, a known GPCR [22]. The remaining LPARs, including LPA_4_-LPA_6_, are structurally distinct from the Edg family and are closely related to the purinergic receptor family (non-Edg family) [23]. Non-Edg family members have a higher affinity for alkyl-LPA species compared to the Edg family members that have higher affinity for the acyl variants [22].

Initial studies suggested that the brain is rich in LPA and LPARs [24–26] and contains enzymes for the synthesis and degradation of LPA [27]. LPA induces numerous responses related to the morphological, pathological, and clinical functions of the CNS [28–38]. The constant level of LPA_1_ expression in undifferentiated and differentiated astrocytes suggests that LPA_1_ primarily mediates the LPA-induced stimulation of DNA synthesis [39]. LPA_1_-LPA_3_ are expressed at extremely low levels in the normal adult brain, but expression is upregulated following brain injury [40]. Following injury or ischemia of the CNS, LPA activity increases in the cerebrospinal fluid [41, 42]. LPA concentrations probably increase in the CNS when the BBB is impaired, including after brain injury, cerebral ischemia, and GBM. LPA_1_, the LPAR responsible for LPA-driven cell motility, is predominantly expressed in GBM [4, 43].

### ATX and GBM

ATX, a 125-kDa glycoprotein, is a multifunctional phosphodiesterase that was originally isolated from melanoma cells as a potent cell motility-stimulating factor [44]. ATX is identical to lysoPLD and catalyzes the production of LPA from lysophosphatidyl choline [18]. ATX not only possesses lysoPLD activity, but it also is a lipid carrier protein that efficiently transports LPA to its receptors, LPA_1_-LPA_6_ [45]. All biological effects of ATX are thought to be attributable to LPA production and subsequent receptor stimulation [46].

ATX is very widely expressed, with mRNA detected in essentially all tissues including high levels of expression in brain [47]. ATX is also present in plasma [9]. ATX is highly expressed in a variety of cancers [48–52] including GBM [53, 54], and is implicated in tumor progression, invasion, and angiogenesis. ATX overexpression in GBM may facilitate invasion and migration through endothelial cells in an autocrine manner, as well as promote neovascularization in the tumor core through paracrine signaling [54].

Most brain cancer cells express high levels of ATX, with the highest expression in the SNB-78 glioblastoma cell line (derived from GBM) [4]. In addition, GBM tissue samples derived from surgical specimens show extremely high ATX expression [4]. GBM may acquire its high invasiveness through autocrine production of LPA by ATX [18]. Inhibition of ATX by its specific inhibitor PF-8380 (Pfizer inflammation research, Missouri, USA) leads to decreased invasion and enhanced radiosensitization of GBM cells [55]. Furthermore, inhibition of ATX leads to diminished tumor vascularity and delayed tumor growth of GBM [55]. As a secreted phosphodiesterase, ATX may be an attractive druggable therapeutic target for GBM.

### Angiogenesis, hypoxia, pseudopalisading necrosis, and LPA

GBM is among the most vascular human tumors [56]. Tumors require angiogenesis to maintain a constant nutrient supply. As the tumor grows, it disrupts pre-existing blood vessels. Newly formed brain tumor blood vessels possess a defective BBB that contributes to the pathogenesis of tumor-associated edema [57], are associated with an increased risk of intratumoral hemorrhage [58], and are responsible for contrast enhancement on computed tomography and magnetic resonance imaging [59].

Intravascular thrombosis within the tumor can clearly accentuate and propagate tumoral hypoxia and necrosis. Intravascular thrombosis within the tumoral tissue of GBM is a frequent intraoperative finding by neurosurgeons [60]. In many instances, intravascular thrombosis is seen within or adjacent to the regions of pseudopalisading necrosis, leading to the proposition that vaso-occlusion due to thrombosis may directly initiate or propagate hypoxia and necrosis in GBM [60]. The plausible contributing factor of intravascular thrombosis in GBM is access of plasma-clotting factors to tumoral tissue. LPA plays a role in regulating platelet function and thrombosis [11]. Plasma ATX is associated with platelets during aggregation and is concentrated in arterial thrombi [61].

Pseudopalisading necrosis has always been a histopathological curiosity in GBM. No other tumor in humans demonstrates such a histopathological alteration [62]. Mamun *et al.* reported that cerebral ischemia/hypoxia promotes rich pseudopalisading necrosis in a rat model of glioblastoma [62] and a middle cerebral artery occlusion modified from a reported method [63, 64]. Vascular occlusion and intravascular thrombosis lead to tissue hypoxia in perivascular regions. The tumor cells then become hypoxic and undergo apoptosis or necrosis, eventually leading to a central necrotic zone [62]. Hypoxic tumor cells produce angiogenic factors, the most predominant of which is vascular endothelial growth factor (VEGF). Hypoxia also induces nuclear accumulation of the hypoxia-inducible factor (HIF) alpha and beta complex, resulting in transcriptional activation of VEGF [65], and can upregulate expression of VEGFR2, a VEGF receptor, in endothelial cells. VEGF signaling contributes to the highly angiogenic nature of GBM [66].

In pathological states, the role of LPA in angiogenesis becomes important [67]. LPA stimulates cancer cell secretion of VEGF and triggers angiogenesis [68]. Lee *et al.* reported that LPA induces VEGF *via* HIF-1 alpha activation [69]. Tissue hypoxia is a critical factor for tumor aggressiveness and metastasis in cancers. HIF-1 alpha plays a critical role in enhancing and/or sensitizing the role of LPA in cell migration and invasion in hypoxic conditions [70]. Both LPA_1_ and LPA_3_ are involved in LPA-induced VEGF secretion [71].

Bevacizumab, a monoclonal antibody that recognizes VEGF, is currently clinically approved for GBM treatment. Unfortunately, although bevacizumab treatment prolongs progression-free survival in a subset of patients, only minimal improvements in overall survival are observed, and patients invariably relapse [72, 73]. This may be explained by several previous observations. As the brain is a highly vascular organ, GBM cells can spread diffusely without necessarily requiring neovascularization [74, 75]. Inhibition of tumor angiogenesis can modulate patterns of tumor invasion [76, 77]. Increasing evidence suggests that anti-angiogenic therapy can lead to enhanced tumor cell invasion [76–79]. Although the exact mechanisms responsible for this increased invasiveness are unknown, researchers have speculated that a decreased supply of oxygen and nutrients may act as a stimulus for tumor cell migration [80]. Lamszus *et al.* reported an interesting double-pronged inhibitory regimen for this condition: a combined treatment directed at VEGFR-2 and the epidermal growth factor receptor (EGFR) [80]. This strategy simulates the ATX/LPA axis-targeted approach. In addition to LPA, ATX plays a role similar to that of VEGF in angiogenesis [81]. Thus, inhibition of the ATX/LPA axis may be beneficial in GBM as a double-pronged therapy that includes anti-angiogenic therapy and anti-invasion therapy.

### Invasion, cell motility, and LPA

Invasion is a defining hallmark of GBM, just as metastasis characterizes other cancers. Drivers of GBM invasion include autocrine signals propagated by secreted factors that signal through receptors on the tumor. Various autocrine motility factors are expressed by invasive GBM cells. Most autocrine and paracrine interactions involved in GBM invasion constitute known signaling systems during CNS development that involve the migration of precursor cells that populate the developing brain [82].

LPA_1_, the LPAR responsible for LPA-driven cell motility, is predominantly expressed in GBM [4]. The pattern of invasion of GBM does not seem to be random, but rather seems to follow the path of blood vessels and more prominently myelinated axons [83]. GBM usually invades along white matter tracts. According to a study using post-mortem human brain tissue, in the normal brain including the cortex and corpus callosum, LPA_1_ is expressed in the white matter along fibers resembling myelinated axons [84]. This is consistent with the presence of LPA_1_ on white matter tracts of adult mouse brains and human cerebral cortex [85]. The LPA_1_ antagonist Ki16425 (Kirin Brewery Co., Takasaki, Japan) effectively suppresses LPA-induced motility of glioblastoma cells. Thus, the motility of these cells appears to depend on ATX and LPA_1_ [4]. LPA_1_-induced cell motility of GBM was also shown in a previous report by Manning *et al.* [43].

Rho family GTPases including Rho and Rac are presumed to modulate various cellular functions such as cytoskeletal reorganization, cell motility, invasion, and proliferation. LPA is especially important in cell adhesion, and LPA signaling has an obvious impact on both focal adhesions (Rho) and lamellopodia (Rac) [86, 87]. Cell motility is tightly controlled by the activities of Rho and Rac in a coordinated manner. The balance of Rho and Rac activities is a critical determinant of cell movement [88]. Each LPAR differentially contributes to these activities.

Recently developed molecular targeted approaches to GBM involving signaling pathways such as EGFR, PDGFR, PI3K/AKT, and RAS predominantly address key pathways involved in cell proliferation, whereas recurrent tumors regrow from the cells that have invaded the brain and may be temporally less proliferative [83]. A novel approach that targets this invasive subpopulation of tumor cells and its environment will be necessary to improve the prognosis of GBM. From this point of view, the new treatment approach targeting LPA and LPAR (LPA_1_) may be promising.

### Subventricular zone (SVZ), neural stem/progenitor cells, and LPAR

Migration is a phenomenon that is mainly present during development [89]. In the adult CNS, the only cells thought to maintain the capacity for motility are stem cells or precursor cells in the subependymal layer that may be recruited for regeneration and repair. Research on human cancers including brain tumors has revealed that tumor stem cells often constitute only a small proportion (<5 %) of the neoplastic cells in these tumors. Most of the cells in the neoplastic stem cell population that are not actively dividing have proven to be quite resistant to commonly used cytotoxic drugs, which preferentially kill rapidly proliferating cells [7].

Cells originating from the stem-cell reservoir have been hypothesized to be a source of glioma cells [90, 91]. Recent evidence suggests that the heterogeneity seen in GBM may be related to the cells of origin, which have stem cell-like characteristics [92–94]. GBM contains a subset of stem-like cells that express the gene for the neural stem cell surface antigen CD133 and are capable of self-renewal, tumor propagation, and differentiation into multiple lineages [93, 95]. This population may play an important role in tumor recurrence because they are resistant to chemotherapy and radiation therapy and are capable of initiating tumors that recapitulate GBM histology [92, 93]. A putative source of glioma cells is the SVZ, the largest area of neurogenesis in the adult human brain [96]. Neural stem cells (multipotent neural progenitor cells) line the lateral ventricles in the SVZ, and recruitment of these progenitor cells may play a role in the aggressive behavior encountered in GBM [97]. In animal studies, the SVZ demonstrates increased susceptibility to tumorigenesis compared with cortical regions [98–100]. Experiments and clinical findings provide evidence that neuronal progenitor cells in the SVZ with a high migratory potential are involved in the aggressive GBM subtype [101]. GBMs that contact the lateral ventricles have been associated with multifocal dissemination [93, 102] and worse overall survival than nonperiventricular GBMs [103]. Jafri *et al.* demonstrated that patients with GBM involving the SVZ have decreased overall survival and progression-free survival, which may have prognostic and therapeutic implications [97]. A comparison of long-term survivors and short-term survivors with GBM showed that tumor location with regard to the SVZ is significantly associated with survival [104].

The LPA_1_ receptor was originally identified from neuronal progenitor cells in the ventricular zone of the developing brain and was initially termed v*entricular zone gene-1* (*vzg-1*) [19]. Human neural progenitors express functional LPARs that regulate cell growth and morphology [105]. CD133(+) stem cells are highly tumorigenic [106] and enriched in recurrent GBM [107]. Lysophospholipids have been reported to regulate a diverse range of stem cell processes including proliferation, survival, differentiation, and migration in adult and embryonic stem cells and progenitors [108]. LPA inhibits neuronal differentiation of neural stem/progenitor cells derived from human embryonic stem cells [109]. Expression of the LPA_1_ receptor is increased in CD133(+) GBM stem cells [110].

Taken together, LPAR (LPA_1_) may be significantly involved in the aggressive behavior and poor prognosis encountered in GBM. Thus, therapies that target LPA_1_ may be potentially beneficial in GBM treatment, especially in preventing invasion, re-growth, and recurrence.

### A novel potential therapeutic approach against GBM

Because LPA is well recognized to be involved in the tumorigenesis and metastasis of a variety of cancers, LPAR antagonists and LPA synthesis inhibitors (ATX inhibitors) have been proposed to be promising drugs for cancer treatment [8]. Almost half of all drugs in current use target members of the GPCR family, making LPARs attractive targets for therapeutic development. Structure-function analysis, molecular modeling, and studies of receptor structure are already contributing to the development of novel receptor-selective antagonists [8]. As previously mentioned, the LPA_1_ antagonist Ki16425 (Kirin Brewery Co., Takasaki, Japan) effectively suppresses the LPA-induced motility of glioblastoma cells [4]. Ki16198 (Kyowa Hakko Kirin Co., Ltd., Tokyo, Japan), which specifically inhibits LPA_1_ and LPA_3_, is a promising orally active LPAR antagonist for inhibiting the invasion and metastasis of pancreatic cancer cells [111].

An LPA_4_ agonist is another possibility. LPA_4_ attenuates LPA_1_-driven migration and invasion, indicating functional antagonism between the two subtypes of LPAR [112]. Thus, an LPA_4_-selective agonist may have some beneficial effects for the treatment of GBM, although the expression levels of LPA_4_ in GBM are relatively low [4]. Although LPA_4_ (P2y_9_/GPR23) was originally isolated from brain, high expression of LPA_4_ is not detected in brain [22]. This may be explained by the observation that specific types of cells in restricted areas express LPA_4_. Rhee *et al.* reported that in an immortalized hippocampal progenitor cell line, high-level expression of LPA_1_ and moderate-level expression of LPA_4_ were detected [113], suggesting that LPA_4_ may affect LPA_1_ activity in brain tumors and/or their environment. To determine the potential role of LPA_1_- and LPA_4_-targeted compounds in GBM, the following questions remain to be answered in future studies. (1) How does LPA_1_ react to various species of LPA within the tumor environment, with and without VEGF inhibition? (2) Is LPA_4_ expressed in neighboring astrocytes or the peri-tumoral environment with regard to the helper nature of mature astrocytes? (3) How will this expression affect the progression of GBM? LPA species with both saturated fatty acids (16:0, 18:0) and unsaturated fatty acids (16:1, 18:1, 18:2, 20:4) have been detected in serum, plasma, and activated platelets [114–116]. Interestingly, these LPA species exhibit different biological activities [117–119], possibly by the differential activation of the different LPARs. For example, LPA with an unsaturated fatty acid induces proliferation and de-differentiation of smooth muscle cells, whereas LPA with a saturated fatty acid does not [118, 119]. These observations clearly indicate the biological significance of LPA species *in vivo*, and they may influence GBM. Whether transactivation of EGFR (by G_12/13_ activation from LPA) may also be suppressed by an LPA_1_ antagonist remains unknown and should be elucidated.

In addition to direct pharmacological modulation of LPARs, several groups have targeted the upstream enzyme ATX for potential therapeutics. As ATX expression accounts for at least half of plasma LPA levels [120], these drugs ultimately attenuate LPA signaling. Several ATX inhibitors have been synthesized. These include the small molecules ONO-8430506 [121], PF-8380 [55], and gintoin, which is a plant-derived LPA/ginseng glycolipoprotein complex that results in feedback inhibition of ATX through LPAR signaling [122]. Whether these inhibitors are reversible may also be an important factor for actual clinical application.

A new type of multi-drug therapy in which several drugs with synergistic effects are administered simultaneously may be beneficial. The potential benefits of targeting ATX/LPAR were shown in murine breast cancer models using a combination of an ATX inhibitor and an LPAR antagonist [123]. Moreover, Schleicher *et al.* reported that BrP-LPA, a novel dual-function pan-LPA antagonist/ATX inhibitor, enhances radiation-induced endothelial cell death, disrupts endothelial cell biological function, and reduces glioma cell viability and migration [124]. BrP-LPA treatment prior to irradiation represses GBM tumor growth *in vivo* [124]. A monoclonal antibody that specifically binds and neutralizes LPA has been developed and is in preclinical development [125, 126].

The predictable potential side effects of these drugs require careful attention. One report has shown that LPA_1_ deficiency leads to a schizophrenia-type pathology in mice [85]. Such devastating side effects must be considered when developing new drugs. Designing new drugs that can retain the desired effects, but not cause any undesirable effects, may be feasible. Currently, no approved drugs targeting ATX/LPAR are available for clinical use. A detailed analysis of the pharmacological properties of synthetic inhibitors, including solubility, toxicity, pharmacokinetics, bioavailability, and permeability will be important in the effort to move these drugs into clinical use [127]. Clinical trials serve as the penultimate step on the path toward clinical use in the treatment of human cancers including GBM [128]. Several LPAR-specific analogues and small molecules have been synthesized. To date, at least three compounds have passed phase I and phase II clinical trials for idiopathic pulmonary fibrosis and systemic sclerosis [129–132]. Although no LPAR-targeting cancer drugs have reached clinical trial stages thus far, pharmaceutical investigation is progressing rapidly [21].

Finally, the author would like to propose a future putative protocol for treating GBM in the following order: (1) Maximum tumor resection using multimodal intraoperative information such as that provided by intraoperative neuroimaging, neuro-navigation, photodynamic diagnosis, neurophysiological monitoring, histology, and possibly photodynamic therapy during surgery; (2) conventional radiation/TMZ therapy; (3) ATX and/or LPAR-targeted therapy instead of TMZ maintenance therapy; and (4) tumor removal or no tumor removal followed by ATX and/or LPAR-targeted therapy in the case of tumor recurrence. Theoretically, this protocol may extend both progression-free survival and overall survival of GBM patients compared to the present standard therapy. The use of LPAR agonists/antagonists and/or ATX inhibitors seems to be an attractive strategy, and such drugs may be promising for the treatment of GBM.

## Conclusion

Therapeutic approaches targeting the ATX-LPA-LRAR cascade may be a realistic addition to the treatment of GBM in the near future.
